# Dynamically accepting and scheduling patients for home healthcare

**DOI:** 10.1007/s10729-017-9428-0

**Published:** 2018-01-05

**Authors:** Mustafa Demirbilek, Juergen Branke, Arne Strauss

**Affiliations:** 1 Warwick Business School, Coventry, UK; 2 Warwick Business School, University of Warwick, Coventry, UK

**Keywords:** Home healthcare, Optimisation, Heuristics, Simulation

## Abstract

The importance of home healthcare is growing rapidly since populations of developed and even developing countries are getting older and the number of hospitals, retirement homes, and medical staff do not increase at the same rate. We consider the Home Healthcare Nurse Scheduling Problem where patients arrive dynamically over time and acceptance and appointment time decisions have to be made as soon as patients arrive. The objective is to maximise the average number of daily visits for a single nurse. For the sake of service continuity, patients have to be visited at the same day and time each week during their episode of care. We propose a new heuristic based on generating several scenarios which include randomly generated and actual requests in the schedule, scheduling new customers with a simple but fast heuristic, and analysing results to decide whether to accept the new patient and at which appointment day/time. We compare our approach with two greedy heuristics from the literature, and empirically demonstrate that it achieves significantly better results compared to these other two methods.

## Introduction

Home Healthcare (HHC), also referred to as in-home care, social care, or domiciliary care, is becoming one of the most important components of healthcare. HHC helps hospitals and retirement homes to free capacity and decrease care delivering cost [1]. The most crucial objective of HHC is to ensure people who need medical attention and daily care to receive high-standard home services. According to patients’ needs, nurses, physicians, doctors, and operators visit patients’ homes periodically and provide services. Many elderly, people who are chronically ill, and individuals with disabilities receive HHC services [2].

In 2005, $53.4 billion were spent on 17,700 HHC service providers in the US according to The National Association for Home Care and Hospice. HHC companies employed 200,000 nurses to service approximately 7.6 million patients in 2007 [3]. Due to some factors such as ageing population, chronic diseases, insufficient capacity of hospitals, etc., it is projected that the demand for HHC doubles by 2030 [4]. The following information shows why HHC is gaining much more importance day by day:

- The number of people aged 65 and over in US will be four times as many by 2040 [5].
- Care of a patient in the home costs only $132 per day whilst $1889 are spent for a patient receiving care in a hospital [3].
- Home-based health technologies cost $3 billion in 2007 versus $7.7 billion in 2012 [1].
- The percentage of American adults who are chronically ill is more than 50% [6].

In this study, we focus on the acceptance decision of a request as well as the scheduling. In the literature, an acceptance policy is occasionally discussed in different areas such as public transportation [7] and vehicle dispatching [8]. The problem starts whenever a patient requests service. The HHC provider has to decide whether or not to accept the patient and, if accepted, assign suitable appointment days and times. Then, for each shift, all nurses start from their homes, visit scheduled patients at the agreed appointment times, serve them for the prescribed time, and finally return to their homes. In this paper, only a single nurse servicing patients in a specific area is considered and any overlaps with other nurses’ regions are ignored. We leave the multi-nurse case to future research. The objective of this problem is to maximise the average number of daily visits performed by the nurse. We require that patients are serviced at the same days and times during their service horizon, which is called consistent, periodic Vehicle Routing Problem (VRP). Furthermore, the problem is dynamic, and acceptance and assignment time decisions have to be made as soon as patients arrive. Although there are some studies providing solutions to this problem by using greedy algorithms in the literature, these algorithms do not consider or only partially consider future demand. We propose a Scenario Based Approach (SBA) which simulates several scenarios, scheduling new customers with a simple but fast heuristic, and analysing results to decide whether to accept the new patient and at which appointment day∖time. The basic idea is to see how many times the new request is assigned amongst all simulated requests and in which time slot it is scheduled most frequently. We examine two different variants for SBA. First, a Daily Scenario Based Approach (DSBA) constructs daily tours based on single day demand. Next, a Weekly Scenario Based Approach (WSBA) constructs weekly tours by taking into account an entire week’s requests simultaneously in each scenario. Our main contributions are the following:

- A new acceptance and scheduling policy based on a solution methodology which anticipates future demand for the Dynamic HHC problem,
- Comparison of two different approaches, one depending on constructing tours for each day of the week independently and the other considering all visits of requests in the week at the same time when constructing tours for each day.
- Comparison of our solution method to two greedy heuristics proposed by Bennett and Erera [9].

In Section 2, we present a literature review related to home health nurse routing and scheduling problems. In Sections 3 and 4, we define the problem and present the solution approaches. In Sections 5, simulation environment, scenarios, and results are presented. Finally, we conclude our study and outline future opportunities in Section 6.

## Literature review

In this section, we go over the most relevant studies in the HHC area and Dynamic Routing Problem (DVRP) since our problem and solution methodology are directly related to the DVRP.

### HHC studies

HHC related models started with Begur et al. [10] in 1997, “An integrated spatial DSS for scheduling and routing home healthcare nurses”. They constructed a decision support system for a home care company to optimise their routing and rostering operation without considering time windows. We refer readers to Mutingi et al. [11] for a state-of-the-art review of the models and algorithms that have been reported in the literature between 1997 and 2013, and concentrate on some key papers from this period.

Gaspero and Urli [12] focused on finding an optimal multi-day HHC schedule by employing a two-stage solution approach. First, they used constraint programming to solve the vehicle routing formulation. Next, they introduced a large neighbourhood search method to improve the initial solution provided by constraint programming. This method was applied to solve a set of random instances that mimic a real-world HHC assignment problem. Experimental outcomes showed that large neighbourhood search significantly improved the constraint programming solution in terms of number of unscheduled patients. However, constraint programming is a better way to reduce the total travelling distance. Bard et al. [13] constructed weekly schedules of HHC staff servicing in 135 nursing homes. They tried to minimise cost over a 5-day planning horizon under over time rules, breaks, and time window constraints. Additionally, preference of patients and nurses were taken into account unless they violated feasibility of the model. They modelled the problem as a large-scale mixed integer programme and used a branch-and-price-and-cut algorithm to solve it. Furthermore, a rolling horizon algorithm was used to find high-quality solutions for larger instances since the branch-and-price-and-cut algorithm was slow to converge. They employed data and regulations such as the practises, policies, legal restrictions, and compensation rules of Key Rehab, a company providing physical, occupational, and speech therapy in US Midwest. Carello and Lanzarone [14] developed a healthcare application based on nurse rostering by taking into account the continuity of care requirement. They used stochastic programming and robust optimisation to model both deterministic and stochastic problem settings without generating scenarios. They tested the algorithm by using real-life data, taken from a HHC service provider in Italy, and observed that the robust approach showed superior results in terms of overtime work and continuity of care compared to non-robust algorithms. However, the algorithms could provide reasonable results for at most a week since computational cost became very high for longer periods. Cappanera and Scutella [15] tried to develop a model that took into account operators’ skill level matching to patients’ needs. They proposed an integer linear programming formulation to solve this assignment problem including scheduling and routing factors. They used real data derived from HHC providers in Italy to evaluate their model and observed that it worked successfully addressing large HHC instances. Duque et al. [16] constructed a decision support system for a social profit organisation that provides HHC in Belgium. They modelled the problem as a bi-objective optimisation model considering two different objectives, satisfying preferences of both nurses and patients and minimising total travel distances. Consistency and periodicity of visits, different visits frequencies depending on patient needs, and caregiver absence were taken into consideration in the model. They suggested a two-stage approach based on the first maximising the most crucial objective, the satisfaction of patients and nurses’ preferences independent of minimising travel distance. At the second stage, the travel distance was minimised with a constraint on worsening the first objective value below a predefined tolerance limit. Zhan et al. [17] studied an HHC routing and appointment scheduling problem with uncertain service times for a doctor. Their objectives are to minimise patients’ waiting times, the doctor’s idle time, and total travel time. First, they solved a small size problem with mixed integer programming under the assumption of known patients’ service time distributions. Next, the problem was modelled as a two-stage stochastic programming problem and the L-Shape method was used since the branch-and-cut algorithm was not able to solve the problem in a reasonable time for larger instances. Finally, they suggested a heuristic method which could calculate approximate cost of idle and waiting times just by considering the predecessor’s random service time. Results showed that the L-Shape method was more efficient than a branch-and-cut algorithm. Additionally, the heuristic provided good results for large size problems. Hiermann et al. [18] considered HHC scheduling problem with nurse-patient preferences, time windows, qualifications, and pre-allocated jobs for a home care company operating in Austria. They aimed to minimise the tour length when considering satisfaction of patients and staff. A two-stage approach was employed to solve the problem. At the first stage, an initial solution was created by constraint programming or random procedure. At the second stage, the initial solution was iteratively optimised by applying one of four meta-heuristics: a memetic algorithm, scatter search, variable neighbourhood search, and simulated annealing. Results showed that all algorithms performed and terminated in reasonable time. Particularly, the memetic algorithm and variable neighbour search provided superior results. Braekers et al. [19] proposed a bi-objective optimisation model to examine the trade-off between operating cost covering overtime and travel costs and service level including preferences of clients and nurses. They solved the problem with a meta-heuristic algorithm based on a multi-directional local search framework. They conducted computational experiments by using several benchmark problem samples generated based on a real data set. The algorithm performed quite well compared to exact solution methods for small size instances. The results showed that allowing for an additional operating cost was able to improve the service level significantly. Mankowska et al. [20] developed a model for daily HHC routing and scheduling. The model covers nurse qualifications, patients’ preferences, interdependent and dependent services where the former requires that some tasks must be handled before other tasks and the latter is taken into consideration when a task needs more than one worker. They aimed at minimising travel and idle times of nurses and providing fair allocation of waiting times amongst the requests. They introduced a mixed integer linear model and solved a small size problem with ILOG Cplex Solver and a large size instance with adaptive variable neighbourhood search algorithm. Tables 1, 2, and 3 represent a classification of publications in terms of objectives/performance measures, constraints, and solution methodologies in the literature.

**Table 1:** A classification of publications in terms of performance measures and objectives

	Travel Time/Cost	Waiting Time/Cost	Patient/Staff Preferences	Unscheduled Patient/Task
Begur et al. [10]	✓			
Gaspero and Urli [12]	✓	✓		✓
Bard et al. [13]	✓	✓		
Carello et al. [14]	✓			
Cappanera et al. [15]	✓			
Duque et al. [16]	✓		✓	
Zhan et al. [17]	✓	✓		
Hiermann et al. [18]	✓		✓	
Braekers et al. [19]	✓	✓	✓	
Bennett and Erera [9]				✓
Mankowska et al. [20]	✓	✓		✓
Our study				✓

**Table 2:** A classification of publications in terms of constraints

	Qualification	Multi worker	Time windows	Consistency/Periodicity	Patient/Staff Preferences	Breaks
	matching					
Begur et al. [10]	✓		✓	✓		
Gaspero and Urli [12]			✓			
Bard et al. [13]			✓		✓	✓
Carello et al. [14]			✓	✓		
Cappanera et al. [15]	✓		✓	✓		
Duque et al. [16]	✓			✓		✓
Zhan et al. [17]			✓			
Hiermann et al. [18]	✓		✓		✓	
Braekers et al. [19]			✓			
Bennett and Erera [9]				✓		
Mankowska et al. [20]	✓	✓	✓			
Our study				✓

**Table 3:** A classification of publications in terms of solution methodologies

	Exact	Heuristics	Single objective	Multi objective	Static	Dynamic
Begur et al. [10]		✓	✓		✓	
Gaspero and Urli [12]		✓	✓		✓	
Bard et al. [13]	✓	✓	✓		✓	
Carello et al.[14]	✓		✓		✓	
Cappanera et al. [15]	✓		✓		✓	
Duque et al. [16]		✓		✓	✓	
Zhan et al. [17]	✓	✓	✓		✓	
Hiermann et al. [18]		✓	✓		✓	
Braekers et al. [19]		✓		✓	✓	
Bennett and Erera [9]		✓	✓			✓
Mankowska et al. [20]	✓	✓	✓		✓	
Our study		✓	✓			✓

As we mentioned above, existing papers in the literature generally focused on static problem settings for which the number of patients was already known, but requests arrive to the system dynamically during service horizon in real life cases. Additionally, they did not consider any acceptance policy. We have found only the study of Bennett and Erera [9] which considers dynamic patient sets. They presented a rolling horizon myopic planning approach for the single nurse HHC problem.

### DVRP studies

In contrast to the classical VRP, real-world applications often force decision makers to design routing plans online where new information becomes available during plan execution. DVRP studies begin with Wilson et al. [21] in 1977. They employed a greedy insertion heuristic to put dynamically arriving requests into a tour for a single vehicle. Interested readers can find detailed literature reviews on the DVRP in [7, 22, 23]. Because the DVRP literature is vast, we only discuss some papers whose solution methods are related to our solution methodology. Ichoua et al. [24] suggested a Tabu Search based solution method to exploit probabilistic knowledge about future request arrivals. They proposed a waiting strategy where vehicles wait at their current locations based on knowledge about future requests if there is a time gap until the next customer service. Hvattum et al. [25] proposed a multi-stage stochastic programming model and a heuristic solution methodology. The heuristic that generated scenarios including scheduled visits and random customers raised from known distributions. Each sample scenario was solved as a deterministic VRP and common features in the sample scenario solutions were employed to construct routes. Bent et al. [26] modelled DVRP with time windows and aimed to maximise the number of daily visits. They proposed a multiple scenario approach based on generating routing plans including both known and future customers. A distinguished plan selected by a consensus function in terms of the smallest travel cost was employed for decision making processes. The multiple scenario approach was tested against greedy approaches under dynamism varying between 30% and 80%. The main difference between the solution methods of Bent and Hvattum et al. is that the multiple scenario approach from [26] works as Tabu Search with adaptive memory by maintaining and updating routing and distinguished plans consisted of current and future customers whilst Hvattum’s heuristic [25] is a multi-stage model in which each stage represents a time interval over the time horizon. The aim is to find a plan that minimises the expected cost of visiting both current and future requests at the beginning of each stage.

Although the problem we consider is certainly related to the dynamic vehicle routing problem, but there are also substantial differences. The typical paper on dynamic VRP considers a single day, and customer requests may arrive whilst vehicles are already under way. The customer requests then have to be integrated into the existing tours, tours can usually be changed dynamically. On the other hand, in our problem we assume all customer requests arrive in the week before the first service, they arrive dynamically, and we have to commit to fixed appointment dates and times for each request when it arrives. Also, whilst usually DVRP problems assume a customer request only has to be serviced once, we assume patients have to be serviced several times a week, over several weeks, and at the same times and days every week.

## Problem definition

The problem we consider is a single nurse HHC scheduling problem in a dynamic environment over a planning horizon.

### Nurse

All patients are visited by a single nurse in a defined geographic service area. Each working day is divided into equally-spaced time slots to schedule patient visits. A set of possible appointment times, *K*, can be defined as:

$$\textit{K=\{b+i\(\phi\) : i = 0,1,...,k\},}$$

where *b* is the earliest time for an appointment and *ϕ* is the time between appointment times. Travel time between patient *i* and *j* is denoted by *m*(*g*_*i*_,*g*_*j*_) in minutes where *g*_*i*_ represents the location of patient *i*. All travel times are always rounded up to the nearest multiple of time slot. Overtime and weekend work are not considered in our model.

### Patients

Inter-arrival times between patients’ requests are exponentially distributed with parameters over the planning horizon. A request *i* from location *g*_*i*_ contains weekly service frequency *f*_*i*_, episode of care *ec*_*i*_ that represents how many weeks patient *i* needs care, service duration for each visit *sd*_*i*_, starting time for the service *K*_*i*_, and weekly allowable visit day combinations. Visits have to be at the same days and times for consecutive weeks during the episode of care.

### Dynamics

The problem is dynamic in that there are many acceptance/rejection decisions during the planning horizon. Thus, the solution depends on our scenarios. At each stage (a request arrives), decisions are whether or not the request is accepted, and if so, which day combination and time slot it should be assigned to. Patients that cannot be scheduled are rejected. We assume that the acceptance/reject decision has to be made straight away (e.g. whilst the patient is still on the phone) and if we reject a patient, the patient will turn to another home healthcare company.

### Constraints

- Let *i* and *j* be two consecutive appointments on a day, and let *g*_*i*_ and *g*_*j*_ represent locations of the patients assigned to those appointments. Every route for that day is feasible, if and only if
  $$K_{i}+sd_{i}+m(g_{i}, g_{j})\leq K_{j}$$
  for any two consecutive appointments, *i* and *j*.
- A task, representing a duty at a patient’s home, has to be carried out as often as determined by its frequency and episode.
- One of the possible day combinations can be selected for each patient.
- Patients, if accepted, must be serviced at same days and times every week during their service horizon.
- A nurse starts a tour from her home and ends the tour at her home again within the shift’s time window.
- A nurse has to handle a task in its scheduled time period.

### Objective

The objective is maximisation of patient visits during the planning horizon. This is different from maximisation of the number of patients served since patients need different numbers of visits. If *T* represents a set of patients accepted over the planning horizon, excluding warm-up, our objective is:

$$\max_{T}\sum\limits_{t \in T}f_{t}ec_{t}.$$

## Solution methods

In this section, we explain two greedy heuristics and our solution methodologies.

### Distance Heuristic

The distance heuristic [9] is a greedy method which assigns a new request between the pair of patients where is the smallest insertion cost/additional travel time. The cost is calculated by subtracting the distance between the predecessor and successor of a request from the sum of distances between the request and its predecessor and successor. If the Euclidean distance between a request and its predecessor and successor are represented as *k*_1_ and *k*_2_ and the Euclidean distance between its predecessor and successor is *k*_3_, the insertion cost, *C* is calculated as:

$$\textit{C}=k_{1}+k_{2}-k_{3}.$$

Therefore, whenever a new patient arrives to the system, the algorithm calculates the cost of insertion of that patient between all requests assigned already in each day of the week if intervals are feasible. After that, the method selects the cheapest interval in a day/days according to visit frequency of the patient. Finally, all visits are scheduled to those cheapest days and time slots during the service horizon of the patient. The appointment time is set according to proximity of the request to its predecessor or successor. For example, if the distance between the request and its predecessor is shorter than than the distance between the request and its successor, the visit will start right after its predecessor visit and enough travel time of course. If there are some days which have the same insertion costs, as a tie-breaker, we assign the visit to one where fewer patient visits are already scheduled to balance the workload of days.

### Capacity heuristic

The distance heuristic schedules appointments next to each other, even if the travel from one appointment to the next requires more than one time unit. In such cases it may be beneficial to allow for a longer time gap between appointments, so that future patients can be inserted in between, without requiring additional travel time.

The capacity heuristic [9] avoids scheduling a new patient directly adjacent to an existing patient if the travel time is longer than a time slot. If a new patient is more than one time slot away from other patients in the schedule, the capacity heuristic assigns it to a time slot which leaves ample time between it and its predecessor and successor patients to be able to assign a future request between them. For example, let us assume that the travel time between a new request and its predecessor (8.00 am) and successor (11.00 am) are 19 and 24 minutes respectively, and service time is 30 minutes for each one. Thus, candidate time slots are 9.00, 9.15, 9.30, 9.45, and 10.00. If we use the distance heuristic, he is scheduled at 9.00 am. In this case, we can schedule at most one additional request at 9.45, 10.00, or 10.15. On the other hand, if we schedule the request at 9.30, there is a possibility to schedule two more patients at 8.45 and 10.15 if they need only one time slot for travelling between their predecessors and successors. Therefore, the capacity heuristic creates gaps for future patients. Of course, there must be enough space between predecessor and successor patients to put the current request into a suitable time slot. If not, requests are assigned as they are assigned with the distance heuristic.

### Scenario based approach

As mentioned in previous sections, distance and capacity heuristics are greedy algorithms which try to choose the best movement whenever a new request arrives without considering or only partially considering future requests. These heuristics accept all requests if they can and ignore that rejecting a request now can allow accepting more requests in the future. Therefore, with SBA, we try to answer two questions. First, do we accept or reject the request? And if we decide to accept the request, which time slot should it be assigned?

The basic idea behind the algorithm is to run a number of simulations (scenarios) and see how many times the request under consideration has been assigned amongst all requests and in which time slot it has been scheduled most frequently. A scenario includes a number of randomly generated requests in terms of the expected demand and expected number of visits as is explained in more detail in the simulation set-up in the next section. We try to make a daily tour with randomly generated requests, previously accepted ones, and the current one by using the cheapest insertion heuristic whose aim is to find the shortest sub-tour. After the tour is full or all requests in the scenario are assigned, we cheque whether the current request has been scheduled and, if so, its time slot.

We study two different variants for SBA. First, the Daily Scenario Based Approach (DSBA) simply constructs daily tours based on daily demand and independent of a request’s multiple visits in the week. Next, the Weekly Scenario Based Approach (WSBA) constructs weekly tours based on one week demand and all visits of the current patient and requests in the scenario.

#### Daily scenario based approach(DSBA)

In DSBA, each day in a week is evaluated separately and independently of other days in the week. Let us illustrate DSBA with an example. Assume that a new request arrives on Monday from a random location in the service area with 3-visit-per-week frequency. Episode of care and service duration do not matter since they are assumed to be the same for all patients. Now we have to decide whether we accept or reject the request.

**Fig. 1:**
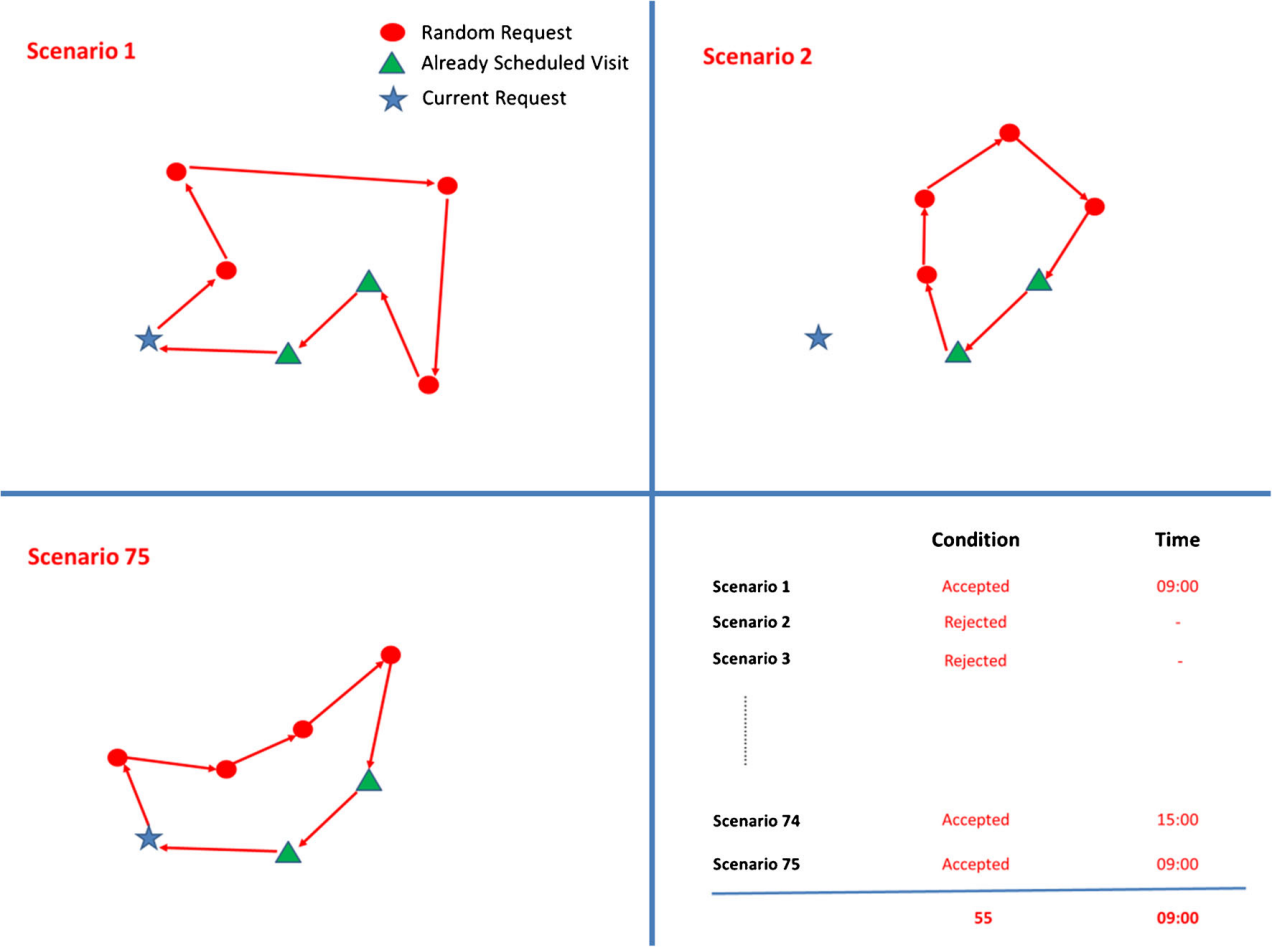
Illustration of generating scenarios and finding the number of acceptance over all scenarios and the most frequent time slot the request is assigned to

First, we generate several scenarios for each day of the next week. Each scenario has a number of randomly generated requests and the current request as shown in Fig. 1. To find how many requests we need to generate randomly, we calculate the average weekly demand. Note that we always calculate a week of demand no matter when a request arrives as explained at the end of this section. If we are looking at next Monday and the expected demand until that day is 10 new patient requests, the total number of visits for next week equals 25 (10*2.5), where 2.5 is the expected weekly visit frequency for a patient. We divide the total number of weekly visits by 5 to determine the average number of visits for a day. It means that 5 requests are generated for each scenario and the current request is added to them.

Next, we try to construct a tour by using requests in the scenario and patients already assigned for that day as illustrated in Fig. 1. Requests are assigned to the tour by using the cheapest insertion heuristic until the tour is full or all requests in the scenario have been scheduled. The cheapest insertion heuristic (CIH) calculates the cost of all possible insertions and finds the one that has the lowest cost.

Once all the requests have been scheduled or no further request can be inserted, we check whether the current request has been scheduled and if so, in which time slot it has been scheduled. After all scenario simulations finish, we find how many times it has been accepted and which time slot it has been assigned to most frequently that day as seen on bottom right Fig. 1. To decide which day combination (Monday-Wednesday-Friday, Tuesday-Thursday-Friday, etc.) it is scheduled, we pick up the best one, two or three days in terms of number of assignments over all scenarios. If the request cannot be scheduled for the number of days that it needs weekly, it is rejected. Algorithm 1 shows the pseudo code for DSBA. ”nReqInTour” in Algorithm 1 represents how many times the request has been scheduled over all scenarios. If it has been assigned at least once, which is called *threshold*, we accept that request. One can see how different thresholds affect the results in Section 5. The number of scenarios is represented by “n” and how to determine the quantity is explained in Section 5.1.2.

We generate random requests based on a week of demand. For example, if a patient arrives on Wednesday, we consider a week demand when checking the next Monday or Friday. However, we have two working days until Monday and seven working days until Friday. The reason to this assumption is that requests that arrive through the end of the week are most likely accepted if the true demand is considered since no other random requests are generated due to the lack of demand. According to our experiments, this set-up outperforms the previous one if the service horizon for patients is only one week. However, if the service horizon is 4-weeks as in our case, the number of daily visits dramatically decreases because accepted patients at the end of the week start blocking acceptance of more suitable requests arriving in subsequent weeks. Therefore, we use a week of demand in all our experiments. 
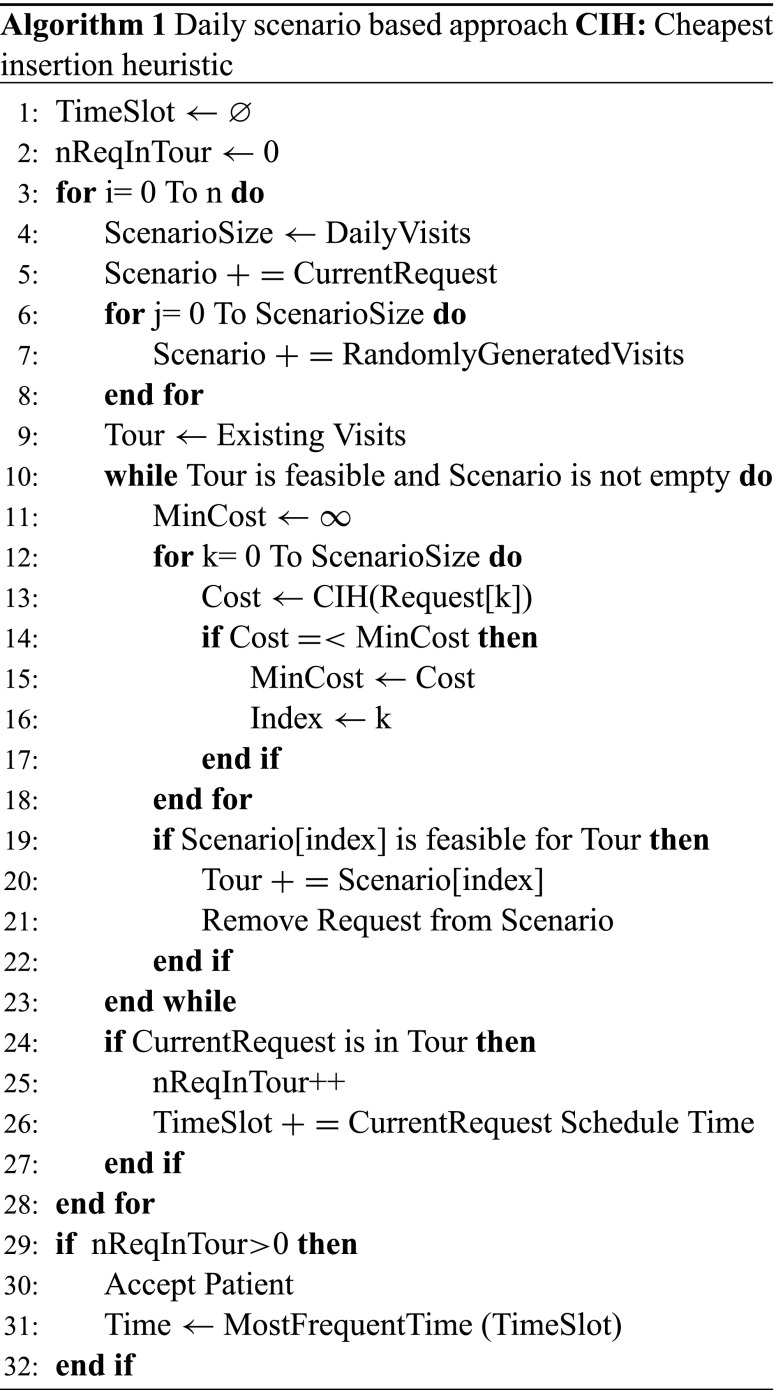


#### Weeky scenario based approach(WSBA)

As explained in the previous section, when generating scenarios for each day in a week, as in DSBA, different visits of the same patient are simply ignored. However, a patient can need 1, 2 or 3 visits in a week and should be considered when generating scenarios for each day of the week. Therefore, we developed a Weekly Scenario Based Approach (WSBA) which constructs tours by taking into account all visits of requests simultaneously in each scenario. In this approach, we generate visits based on expected weekly demand and weekly visit frequency of patients, and construct a weekly schedule with corresponding daily tours by using the cheapest insertion heuristic until the tour is full or all requests in the scenario have been scheduled. After repeating the same process for several scenarios, we choose the day combination to which the current request has been most frequently assigned over all scenarios. Patients who can not be scheduled in any scenario are rejected. Algorithm 2 shows the pseudo code for WSBA. “nCombinations” represents the days for which visits of a patient can be scheduled. As an illustration, assume that there are 5 randomly generated requests (R1 to R5) with different weekly visits and Request A which is under consideration whether to accept or not in the scenario. Table 4 represents these requests with the number of visits they need and insertion cost in terms of travel times for each day. The insertion cost for each day is calculated as we do in DSBA. The algorithm selects the cheapest day/days depending on the number of visits that a request needs. Summing up cost of those days gives the total cost. Monday and Wednesday results in the cheapest total cost for R4 whilst R1 should be assigned to Monday since it needs only one visit and the cheapest insertion cost is computed for Monday. Lines 15-19 in Algorithm 2 show the calculation of the cheapest day set as shown in the example above. Table 5 shows iterations where the algorithm compares requests in the scenario and selects the cheapest in terms of the average cost. The average cost is computed in order to compare insertion costs of patients who need different numbers of visits. R2 is chosen and removed from the scenario in the first iteration in Table 5. In the second iteration, the total costs for all remaining requests are recalculated as in Table 4 and Request A is selected and removed from the scenario this time due to having the smallest average cost. These iterations last until no request remains in the scenario or the tour becomes full. As can be seen in the next section, we use two different day sets, day set 1 and 2. The former allows all possible day combinations whereas the latter only allows specific day combinations. When testing WSBA, we employ day set 2 since the computational time is linear with the possible number of day combinations. Assignments of visits at the same time in the scenario and comparison of average assignment cost distinguish WSBA from DSBA.

**Table 4:** Assignment cost for each visit of requests and total cost

	Visit	Monday	Tuesday	Wednesday	Thursday	Friday	Day set	Total cost
R1	1	50	60	55	80	80	Mon	50
R2	3	30	...	40	...	20	Mon-Wed-Fri	90
R3	3	50	...	30	...	40	Mon-Wed-Fri	120
R4	2	50	60	50	80	60	Mon-Wed	100
R5	2	80	40	50	40	70	Tue-Thu	80
A	3	70	...	50	...	70	Mon-Wed-Fri	190

**Table 5:** Selection of requests

	Iteration 1			Iteration 2		Iteration 3	
	Visit	Total cost	Average cost	Total cost	Average cost	Total cost	Average cost
R1	1	50	50	60	60	30	30
R2	3	90	**30**	...	...	...	...
R3	3	120	40	150	50	120	40
R4	2	100	50	120	60	140	70
R5	2	80	40	150	50	100	50
A	3	190	63	100	**33**	...	...


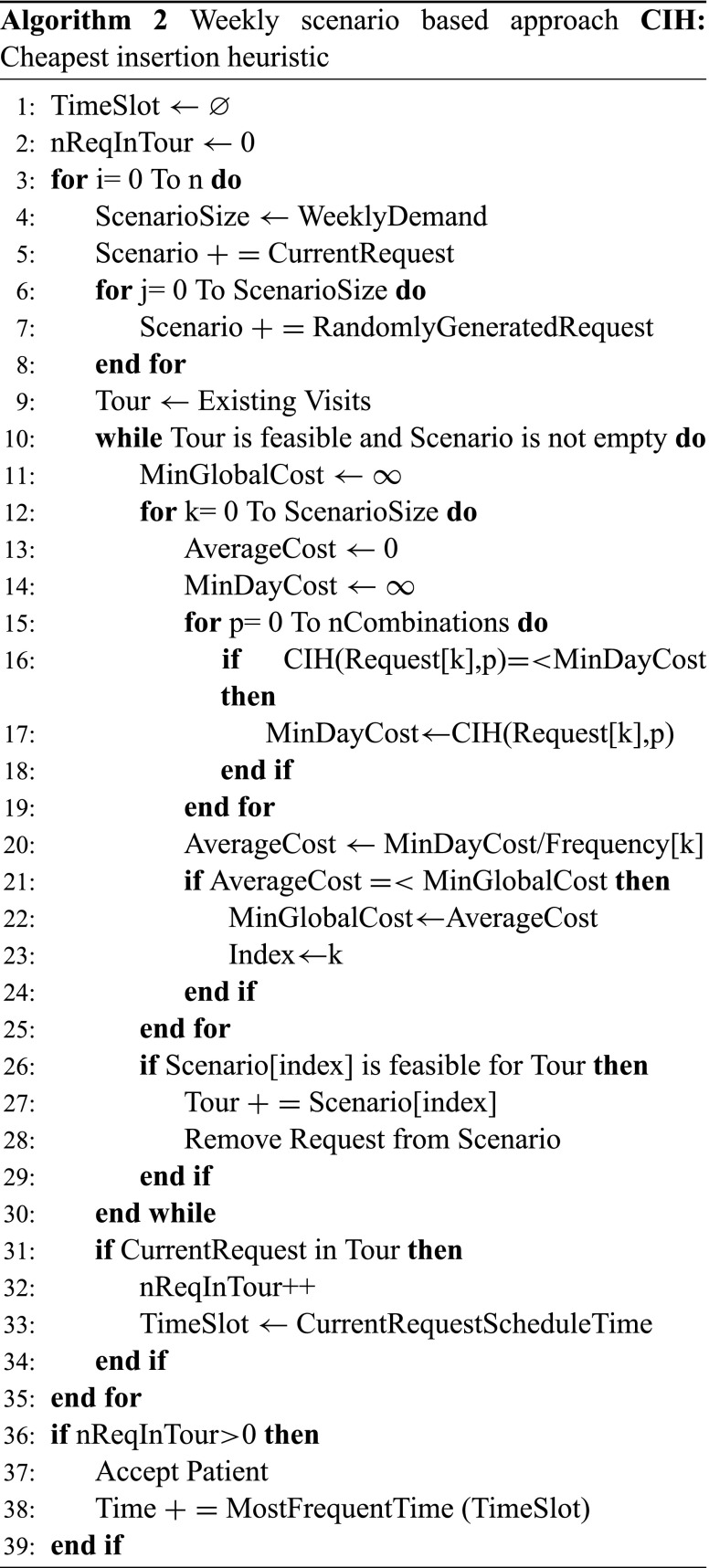


## Simulation and results

### Experimental set-up

We run 30 simulations for each experiment. Each simulation horizon is 360 working days where each day lasts 510 minutes. A day is composed of 34 time slots (*k = 34*) which last 15 minutes. The nurse works between 08.00 and 16.30 each day during the planning horizon. 20-day warm-up period is set at the beginning of the simulation. Inter-arrival times between requests are exponentially distributed with mean 510, 340, or 255 minutes (we have three trials). This corresponds to approximately 1, 1.5, and 2 requests per day, respectively. Each patient has to be serviced over 4 weeks with stochastic visit frequency 1, 2, or 3 visits per week with probabilities 0.05, 0.35, and 0.60, respectively. The first visit is scheduled in the following week after the request has been accepted. Visit durations are deterministic and take 30 minutes. Each arriving customer request is equally likely to arise from either a small square geographic region subdivided into 900 equally-sized square subregions or a large square geographic region subdivided into 3,600 equally-sized square subregions. The reason of using two different area sizes is to observe how algorithms react to short and long travel times. Simulation parameters are shown in Table 6. The nurse (depot) is located in the centre of both regions. To understand differences between simulation results, we conduct independent samples t-tests. Numbers written in bold font mean that they are statistically different. We have two different set-ups for visit days each patient can be assigned to according to his weekly visit frequency. In the first set-up, each patient can be scheduled any combination of days in the week. Because we do not allow weekend work, there are $\binom {n}{f}$ day combinations for a patient where *f* represents the weekly visit frequency and *n* represents the number of days (Monday, Tuesday, Wednesday, Thursday, Friday). This is called day set 1. Although most studies in the literature allow any day combinations when assigning requests, some authors [16] emphasises not to use sequential days if multiple visits are taken into consideration. Intuitively, it does not make sense to perform some tasks such as cooking, bathing, etc. the first two or three days at the beginning of a week and to do nothing at the remaining days when considering real life cases. Thus, we also use another day set-up which does not allow to schedule sequential days when the visit frequency of a patient is two or three. Therefore, only the following visit day combinations can be assigned to a patient who needs two visits in a week, *((Monday,Friday),(Monday,Thursday), (Tuesday,Friday),(Tuesday,Thursday))* and a patient who needs three visits in a week, *(Monday,Wednesday,Friday)*. This set-up is called day set 2.

**Table 6:** Simulation setup

Simulation parameters		
Simulation horizon (day)		360
Warm-up period (day)		20
Daily working time (minute)		510
Service Horizon (week)		4
Interarrival times (minute)		510,340,255
Weekly visit frequency		1,2,3
Weekly visit probability		0.05,0.35,0.60
Small area (*X*_1_, *X*_2_, *Y*_1_, *Y*_2_)		0,30,0,30
Large area (*X*_1_, *X*_2_, *Y*_1_, *Y*_2_)		0,60,0,60

Note that our approach does not depend on above parameter settings such as time intervals, appointment durations, service horizon, or non-uniform demand. It can be applicable for different parameter setting as well.

#### Determination of scenario size

**Fig. 2:**
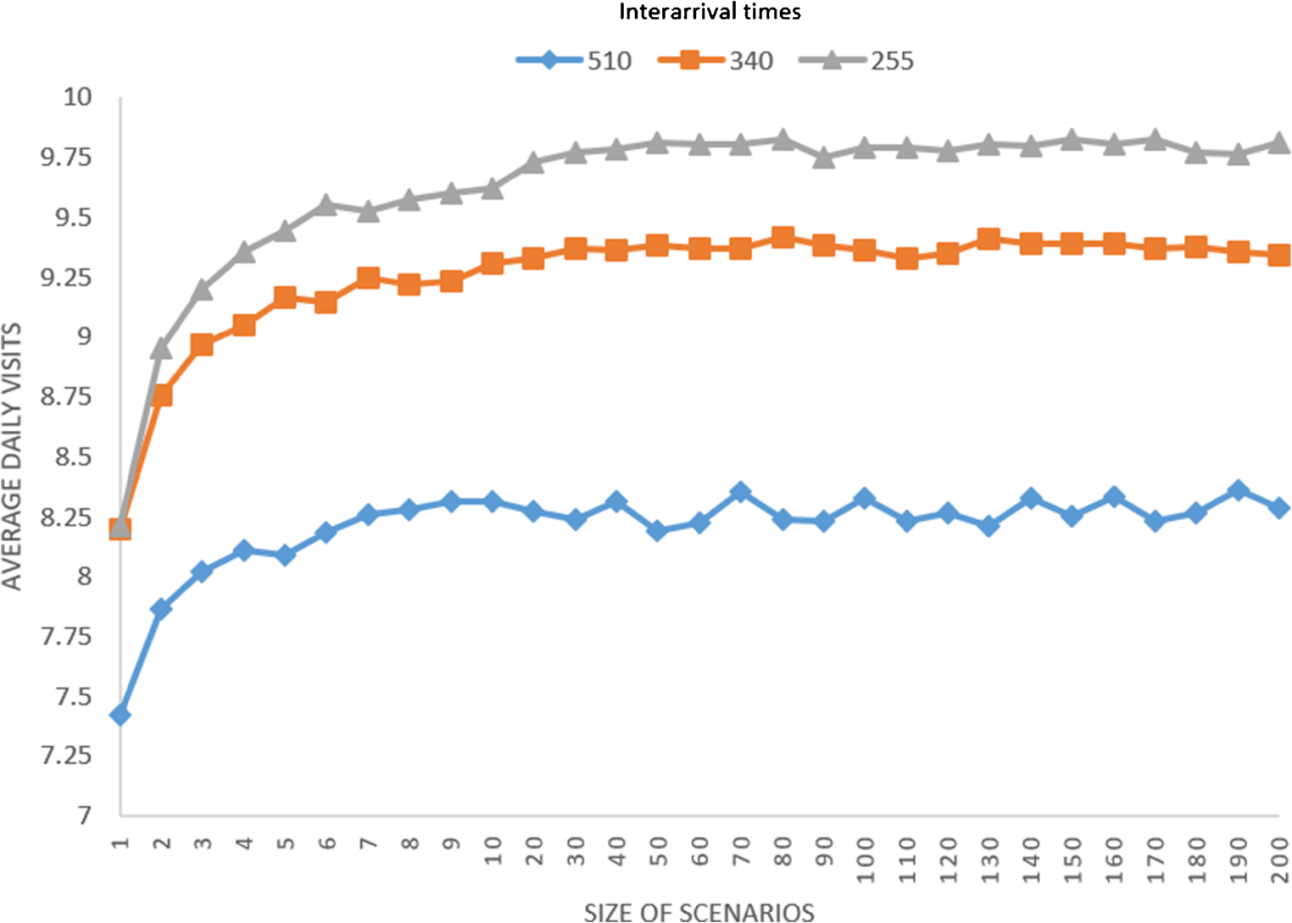
Average daily visits under different scenario sizes and inter-arrival times

In DSBA and WSBA, we fixed the scenario size to 75. Obviously, a large number of scenarios means longer computational time. On the other hand, a lower size of scenarios can cause decreasing quality of estimation for appointment times. Therefore, we tried different numbers of scenarios to observe how it affects results. Figure 2 shows the average number of daily visits under different scenario sizes and inter-arrival times for day set 1, a small region, and the predefined experimental setting. The results for the three different inter-arrival times stabilise at scenario sizes above 70 or 80. To further increases the number of scenarios rises the computational cost but no longer improves the quality of solution.

#### Determination of acceptance threshold

**Fig. 3:**
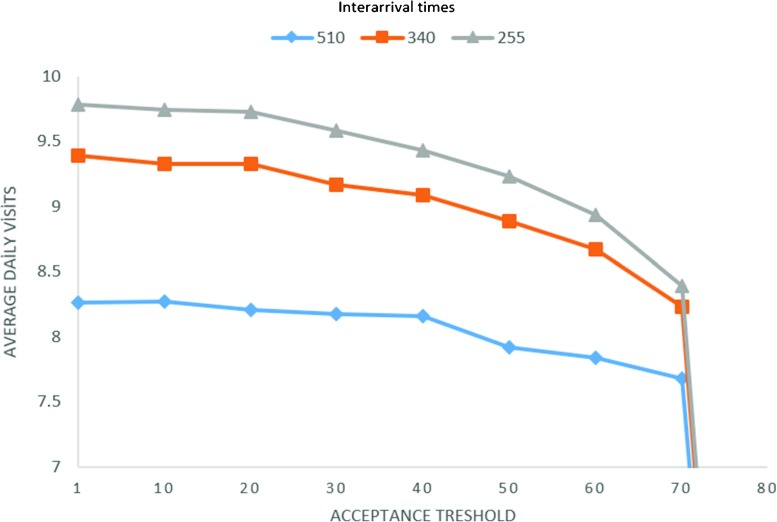
Average daily visits for different acceptance thresholds

As we mentioned previously, one of our aims in this study is to develop an acceptance policy. We believe that rejection of some patients helps to accept more patients in the future. In DSBA and WSBA, some scenarios are generated and daily/weekly tours are constructed. The purpose is to check whether or not the current request should be accepted. However, how many times across the number of scenarios should a patient be assigned to be able to accept it? To determine the setting, we tried different acceptance thresholds and results are reported in Fig. 3. Again we conducted three trials for different inter-arrival times and the same experimental setting was used as in Section 5.1.1. Figure 3 clearly demonstrates that average daily visits tend to reduce when the acceptance threshold is increased. The reason is that accepting a request is getting harder when we increase the threshold. Particularly, if the demand is high, the decline of average number of daily visits is steeper since a high threshold decreases the probability of acceptance. Therefore, we fixed the acceptance threshold to 1. It means that we accept a patient if he/she can be scheduled at least once over 75 scenarios.

Note that always accepting the patient would be similar to DH and leads to inferior results.

### WSBA vs DSBA

In this section, we compare the two different solution methodologies that we developed, WSBA and DSBA. As explained in the previous section, the main difference between the two methodologies is that each tour constructed for a day is independent of the remaining days in the week in DSBA. On the other hand, weekly tours are constructed by using visits belonging to the same requests in WSBA. The latter is more realistic since requests need one, two, or three visits in a week and generating different requests for each scenario without considering these visits as in DSBA can affect the results. However, Tables 7 and 8 show that results of average daily visits, travel times per person, and acceptance rates are close. None is superior to the other since DSBA provides slightly better results in some cases whilst there are other cases where WSBA works better. Because our objective is to maximise average number of daily visits, it is more important to look at results of visits for WSBA and DSBA. As can be seen in the first three columns in Table 7, DSBA results are slightly higher for the small region, but only the difference between average number of visits for WSBA and DSBA under large area and the high demand scenario is statistically significant.

**Table 7:** Comparisons of WSBA and DSBA in terms of average number of daily visits, travel times per person, and patient acceptance rate for the small region

	**Times** ^∗^	**WSBA**	**DSBA**
Daily visits	510	6.97 ± 0.05^∗∗^	7.00 ± 0.04
	340	8.07 ± 0.04	8.09 ± 0.03
	255	8.61 ± 0.02	8.65 ± 0.03
Travel times	510	**16.67**± 0.18	**17.36**± 0.15
	340	**15.35**± 0.09	**15.64**± 0.08
	255	**14.18**± 0.08	**13.92**± 0.07
Acceptance rate	510	0.72 ± 0.004	0.73 ± 0.005
	340	0.58 ± 0.003	0.59 ± 0.003
	255	0.49 ± 0.003	0.49 ± 0.004

^∗^Under consideration of 510-minute day length, interarrival times result in approximately 1, 1.5, and 2 requests per day, respectively.

^∗∗^ Standard error

**Table 8:** Comparisons of WSBA and DSBA in terms of average number of daily visits, travel times per person, and patient acceptance rate for the large region

	**Times**	**WSBA**	**DSBA**
Daily visits	510	6.05 ± 0.04	6.08 ± 0.03
	340	6.81 ± 0.03	6.81 ± 0.01
	255	**7.28**± 0.02	**7.18**± 0.02
Travel times	510	**28.58**± 0.24	**29.43**± 0.17
	340	**26.66**± 0.13	**25.34**± 0.10
	255	**24.75**± 0.20	**24.03**± 0.14
Acceptance rate	510	0.51 ± 0.005	0.51 ± 0.004
	340	0.64 ± 0.004	0.65 ± 0.002
	255	0.41 ± 0.003	0.41 ± 0.002

Computational cost is a crucial factor for this study since a decision has to be made as soon as someone requests a service. Therefore, execution times are reported for WSBA, DSBA, DH (Distance Heuristic), and CH (Capacity Heuristic) in Table 9. Each time is measured during a trial in which day set 2 is used. CH and DH need relatively short times compared to our methodology. Execution times for WSBA are significantly longer than DSBA execution times even though we use day set 2 for the trial. It is clear that assessing a whole week with all visits of different requests in each scenario of WSBA significantly increases computations compared to decomposing a week into separate days and evaluating them independently in DSBA. We decided to use DBSA since results are close and the computational cost of DSBA is much lower than WSBA’s.

**Table 9:** Execution times for each method(millisecond)

**Method**	**510**	**340**	**255**
**WSBA**	24,927	78,676	177,489
**DSBA**	1,741	2,813	6,873
**CH**	33	47	56
**DH**	32	42	51

Note that WSBA is conceptually more appropriate than DBSA, as it simultaneously looks at all appointments required by a patient over the week. But it is computationally much more expensive, and the additional benefit in terms of performance in the scenarios considered in our study is marginal. However, for problems with more interdependence between the days, WSBA may have advantages.

### DSBA, distance, and capacity heuristics

Table 10 shows the average number of daily visits according to DH, CH, and DSBA. Our methodology yields superior results for both small and large regions and different inter-arrival times. Particularly, daily visits increases substantially compared to DH and CH in the small region if demand is relatively high. Average number of daily visits by using DH is higher than by using CH in the large area, and the improvement by SBA reaches around 11% and 6% compared to CH and DH. All results are significantly different from each other.

**Table 10:** Average daily visits for DH, CH, and DSBA by using day set 1

**Region**	**Times**	**DH**	**DSBA**	%	**CH**	**DSBA**	%
Small	510	**8.19**± 0.02	**8.31**± 0.03	**1.46**	**8.21**± 0.02	**8.31**± 0.03	**1.32**
Small	340	**9.03**± 0.02	**9.38**± 0.03	**3.87**	**9.14**± 0.03	**9.38**± 0.03	**2.61**
Small	255	**9.28**± 0.02	**9.79**± 0.02	**5.50**	**9.49**± 0.02	**9.79**± 0.02	**3.22**
Large	510	**6.97**± 0.02	**7.09**± 0.02	**1.81**	**6.57**± 0.01	**7.09**± 0.02	**7.98**
Large	340	**7.54**± 0.02	**7.88**± 0.02	**4.49**	**7.18**± 0.02	**7.88**± 0.02	**9.68**
Large	255	**7.79**± 0.02	**8.26**± 0.02	**5.97**	**7.46**± 0.02	**8.26**± 0.02	**10.75**

Table 11 demonstrates travel times per visit for the three approaches. DH and CH provide shorter travel times than DSBA since it does not benefit from its ability to select more suitable requests under low demand. When demand is higher, DSBA also ensures travel times at least as good as DH and CH or better even though number of patients serviced is more than for the other two methods. Particularly, travel times in SBA are significantly lower in a large area and when demand is medium and high.

**Table 11:** Average travel time per visit for DH, CH, and DSBA (minute) by using day set 1

**Region**	**Times**	**DH**	**DSBA**	%	**CH**	**DSBA**	%
Small	510	14.75 ± 0.04	**15.46**± 0.05	4.76	**14.92**± 0.05	**15.46**± 0.04	3.57
Small	340	**15.24**± 0.06	**14.68**± 0.07	**-3.72**	**15.07**± 0.07	**14.68**± 0.07	**-2.62**
Small	255	**14.98**± 0.05	**13.68**± 0.08	**-8.68**	**14.88**± 0.08	**13.68**± 0.08	**-8.05**
Large	510	**26.63**± 0.09	**25.87**± 0.08	**-2.86**	**26.83**± 0.08	**25.87**± 0.08	**-3.58**
Large	340	**26.17**± 0.15	**24.42**± 0.12	**-6.70**	**26.64**± 0.14	**24.42**± 0.12	**-8.35**
Large	255	**25.75**± 0.14	**22.63**± 0.14	**-12.11**	**26.07**± 0.13	**22.63**± 0.14	**-13.19**

Table 12 represents acceptance rates (number of accepted requests/total requests) for the three methods. Although DH and CH accept all they can and do not reject any request if they have an available place for it, acceptance rates of our methodology are higher in all experiments. This shows that rejection of some requests now can help to accept more requests overall in the future. In a small region, acceptance rates are close to each other because of low demand condition. The proposed methodology takes demand fluctuation into account and accepts as many patients as possible if the demand is low. However, it can be seen that our methodology significantly increases acceptance rates under scenarios of small region-high demands and large region. As for the results of average number of daily visits, results of travel times per patient and acceptance rates are statistically significantly better.

**Table 12:** Acceptance Rates for DH, CH, and DSBA by using day set 1

**Region**	**Times**	**DH**	**DSBA**	%	**CH**	**DSBA**	%
Small	510	**0.81**± 0.003	**0.83**± 0.003	**2.34**	**0.82**± 0.003	**0.83**± 0.003	**1.73**
Small	340	**0.62**± 0.004	**0.65**± 0.004	**4.63**	**0.63**± 0.005	**0.65**± 0.004	**3.18**
Small	255	**0.48**± 0.004	**0.53**± 0.003	**8.50**	**0.49**± 0.003	**0.53**± 0.003	**7.16**
Large	510	**0.71**± 0.003	**0.72**± 0.004	**1.24**	**0.67**± 0.002	**0.72**± 0.004	**6.78**
Large	340	**0.52**± 0.004	**0.55**± 0.004	**5.82**	**0.50**± 0.004	**0.55**± 0.004	**9.68**
Large	255	**0.41**± 0.002	**0.45**± 0.003	**9.39**	**0.40**± 0.003	**0.45**± 0.003	**13.62**

Tables 13, 14 and 15 represent the results of average number of daily visits, travel times per person, and acceptance rates for DH, CH, and DSBA for day set 2. DSBA provides higher average daily visits and lower travel times per person for both small and large regions and different inter-arrival times. All differences of average daily visits and travel times are statistically significant. Particularly, average number of daily visits provided by DSBA tend to increase when the demand is low. On the other hand, saving travel times per person is going up when areas become bigger and demand increases for DSBA. However, acceptance rates are not statistically significantly different from each other in some cases.

**Table 13:** Average daily visits for DH, CH, and DSBA by using day set 2

**Region**	**Times**	**DH**	**DSBA**	%	**CH**	**DSBA**	%
Small	510	**6.52**± 0.02	**7.00**± 0.04	7.4	**6.63**± 0.04	**7.00**± 0.04	**5.6**
Small	340	**7.80**± 0.02	**8.09**± 0.03	**3.7**	**7.85**± 0.03	**8.09**± 0.03	**3.1**
Small	255	**8.29**± 0.03	**8.65**± 0.03	**4.3**	**8.51**± 0.03	**8.65**± 0.03	**1.6**
Large	510	**5.9**± 0.01	**6.08**± 0.01	**3.1**	**5.52**± 0.02	**6.08**± 0.01	**10.2**
Large	340	**6.69**± 0.04	**6.81**± 0.03	**1.9**	**6.32**± 0.04	**6.81**± 0.03	**7.8**
Large	255	**7.06**± 0.02	**7.18**± 0.02	**1.7**	**6.73**± 0.03	**7.18**± 0.02	**6.7**

**Table 14:** Average travel time per visit for DH, CH, and DSBA (minute) by using day set 2

**Region**	**Times**	**DH**	**DSBA**	%	**CH**	**DSBA**	%
Small	510	**18.88**± 0.11	**17.36**± 0.15	**-8.6**	**18.15**± 0.13	**17.36**± 0.15	**-4.4**
Small	340	**16.67**± 0.10	**15.64**± 0.09	**-6.32**	**16.82**± 0.07	**15.64**± 0.09	**-7.5**
Small	255	**16.35**± 0.10	**13.92**± 0.06	**-14.9**	**16.11**± 0.09	**13.92**± 0.06	**-13.4**
Large	510	**30.95**± 0.22	**29.27**± 0.17	**-5.4**	**32.22**± 0.26	**29.27**± 0.17	**-9.2**
Large	340	**27.40**± 0.08	**25.34**± 0.10	**-7.5**	**28.50**± 0.08	**25.34**± 0.10	**-11.1**
Large	255	**28.46**± 0.16	**24.03**± 0.14	**-15.6**	**29.23**± 0.20	**24.03**± 0.14	**-17.8**

**Table 15:** Acceptance Rates for DH, CH, and DSBA by using day set 2

**Region**	**Times**	**DH**	**DSBA**	%	**CH**	**DSBA**	%
Small	510	**0.68**± 0.003	**0.73**± 0.005	**7.4**	**0.71**± 0.005	**0.73**± 0.005	**2.8**
Small	340	0.60 ± 0.003	0.60 ± 0.003	0	0.59 ± 0.003	0.60 ± 0.003	1.7
Small	255	**0.48**± 0.004	**0.49**± 0.004	**2.1**	**0.51**± 0.003	**0.49**± 0.004	**4.1**
Large	510	0.65 ± 0.004	0.65 ± 0.004	0	**0.62**± 0.006	**0.65**± 0.004	**4.8**
Large	340	0.53 ± 0.002	0.54 ± 0.002	1.9	**0.50**± 0.002	**0.54**± 0.002	**8**
Large	255	**0.44**± 0.002	**0.43**± 0.003	-2.3	**0.42**± 0.003	**0.43**± 0.003	**2.3**

## Conclusion and future work

Because of increasing average life expectancy, chronic diseases, and insufficiency of healthcare facilities, home care is getting more and more crucial everyday. However, many people who need care cannot access home care services due to lack of care workers. Therefore, companies have to use their workers’ time efficiently in the scheduling and routing process.

In this study, the problem is dynamic and assignment time decisions have to be made as soon as patients arrive by considering service continuity. There is only one study in the literature providing solutions to this problem and it suggested greedy algorithms. We propose a Scenario Based Approach (SBA) which is based on generating several scenarios of future demand to see whether or not we can assign visits of the patient who is currently under consideration. If we can, we check how many times and which time slots most frequently the patient is scheduled over all scenarios. Otherwise, the patient will be rejected.

We develop and analyse two different approaches, Daily SBA (DSBA) and Weekly SBA (WSBA). The former generates scenarios based on daily demand whereas the latter generates scenarios based on generation visits based on weekly demand and visit frequency of patients. In the considered problem instances, the results are similar whilst the computational time for WSBA is significantly higher than DSBA’s. Therefore, we test and compare DSBA to the distance and capacity heuristics. We construct a simulation model where requests arrive according to a Poisson distribution. We make 6 trials based on two differently sized regions and 3 different inter-arrival times. DSBA is clearly superior to distance and capacity heuristics in each scenario based on the average number of daily visits and patient acceptance rates. The travel times of our method are slightly higher under low-demand scenarios whilst DSBA provides significantly shorter travel times at medium and high demands and larger areas. Particularly, we have significant improvements compared to the other two methods under 1.5 and 2 requests per day for most of cases. DSBA increases average daily visits by up to 10% and lessens travel times per patient by up to 13% compared to the distance and capacity heuristics. Additionally, we also test our algorithm only if special day combinations are allowed for multiple visits. Results show that DSBA provides up to 7.5% and 10% higher daily visits and up to 17% lower travel times compared to the the distance and capacity heuristics.

The most important advantage of our application from the perspective of practitioners is that they will be capable of assessing and answering a request quickly without waiting until the beginning of the next schedule period.

In this study, the route of a single HHC nurse is optimised for dynamic patient sets. Whilst this paper deals with the simplest case of a single nurse, in practise many HHC providers will employ multiple nurses with different skills. In future research, we plan to extend our study to such cases. One challenge is that continuity of care will require a patient to be serviced always by the same nurse, and some preliminary tests have already shown that this is difficult to achieve with DSBA, and that WSBA has clear advantages in case of multiple nurses. Furthermore, deterministic travel and service times seem strong assumptions and it seems worth considering stochastic travel and service times as well.
